# Modulation Peroxisome Proliferators Activated Receptor alpha (PPAR α) and Acyl Coenzyme A: Cholesterol Acyltransferase1 (ACAT1) Gene expression by Fatty Acids in Foam cell

**DOI:** 10.1186/1476-511X-8-38

**Published:** 2009-09-02

**Authors:** Javad Zavvar Reza, Mahmoud Doosti, Masoud salehipour, Malehieh PackneJad, Majed Mojarrad, Mansour Heidari, Effat S Emamian

**Affiliations:** 1 Department of Medical Biochemistry, Faculty of medicine, Tehran University of Medical Scenses, Tehran, Iran; 2 Department of Medical Biochemistry, Faculty of medicine, shahid sadoughi University of Medical Sciences, Yazd , Iran; 3 Department of Medical Genetics, Faculty of medicine, Tehran University of Medical Scenses, Tehran, Iran; 4 Advanced Technologies for novel therapeutics, 211 warren street, Newark, NJ, 07103, USA

## Abstract

**Background:**

One of the most important factors in the initiation and progression of atherosclerosis is the default in macrophage cholesterol homeostasis. Many genes and transcription factors such as Peroxisome Proliferators Activated Receptors (PPARs) and Acyl Coenzyme A: Cholesterol Acyltransferase1 (ACAT1) are involved in cholesterol homeostasis. Fatty Acids are important ligands of PPARα and the concentration of them can effect expression of ACAT1. So this study designed to clarified on the role of these genes and fatty acids on the lipid metabolism in foam cells.

**Methods:**

This study examined effects of c9, t11-Conjugated Linoleic Acid(c9, t11-CLA), Alpha Linolenic Acid (LA), Eicosapentaenoic Acid (EPA) on the PPARα and ACAT1 genes expression by using Real time PCR and cholesterol homeostasis in THP-1 macrophages derived foam cells.

**Results:**

Incubation of c9, t11-CLA, LA cause a significant reduction in intracellular Total Cholesterol, Free Cholesterol, cellular and Estrified Cholesterol concentrations (*P *≤ 0.05). CLA and LA had no significant effect on the mRNA levels of ACAT1, but EPA increased ACAT1 mRNA expression (*P *= 0.003). Treatment with EPA increased PPARα mRNA levels (*P *≤ 0.001), although CLA, LA had no significant effect on PPARα mRNA expression.

**Conclusion:**

In conclusion, it seems that different fatty acids have different effects on gene expression and lipid metabolism and for complete conception study of the genes involved in lipid metabolism in foam cell all at once maybe is benefit.

## Background

Between factors in the atherosclerosis, macrophages have the most important role. These cells uptake modified low density lipoproteins (m-LDL) particles such as oxidized LDL (ox-LDL) by scavenger receptors, that leads esterified cholesterol (EC) deposition in the cytoplasm and promote a local pro-inflammatory response [1]. EC stored in the cytoplasm is in a equilibrium with free cholesterol (FC) that can leave the cell. Imbalanced in this equilibrium can convert a macrophage to the a foam cell. In the cholesterol loading macrophages (foam cells), cholesterol turnover intra foam cells is closely related to expression of many genes. Alteration in rate of expression these genes effect the macrophage cholesterol influx and efflux, a very important factor in the initiation and progression of atherosclerosis. Between of nuclear receptor families that can bind fatty acids, only PPAR is completely accepted as a fatty acid-regulated nuclear receptor. Many studies showed that Proxisome Proliferators Activator Receptors (PPARs) represent important regulators of genes involved in macrophage and foam cholesterol homeostasis [2]. Three PPAR subtypes (*α*, *β*, *γ*, and *δ*) have been described that two members of this family, PPAR α and PPAR γ, have critical role in regulation of glucose and lipid metabolism [2]. PPARα is expressed in many tissues such as liver [3], kidney [4], heart and also in cells of the arterial wall, in monocytes/macrophages [5], smooth muscle cells [6], and endothelial cells [7,8]. PPARα activates expression of genes involved in fatty acids catabolism [9], ketone bodies synthesis [10], insulin effects [11] and lipoprotein assembly [12].

Fatty Acids and Synthetic ligands can bind PPAR subtypes and activate then [13], so these compounds in the management and treatment of atherosclerosis can used [14].

Saturated and unsaturated fatty acids of ≥ 14-carbon especially 18- and 20-carbon fatty acids are likely preferred ligands [15]. Two enzymes directly involved in the homeostasis of FC and EC. When cellular free cholesterol level decreased, Cholesteryl Ester Hydrolase(CEH) convert cholesterol esters to free cholesterol and fatty aids[16]. In contrast to the function of CEH, enzyme Acylco A: Cholesterol Acyltransferase (ACAT) catalyzed biosynthesis of EC from free cholesterol and long chain fatty and located in the rough endoplasmic reticulum (RER). This change made cholesterol less soluble in the cytosol and facilitating its storage in cytoplasmic lipid droplets as EC [16]. Two isofoms of ACAT have been described. ACAT1 is ubiquitous, while ACAT2 is expressed primarily in the liver and intestine of humans and animals. The main function of ACAT1 is to prevent excess FC within cell and cell membrane; in contrast ACAT2 has a role in lipoprotein synthesis and secretion in liver and cholesterol absorption in intestine [17]. The level of ACAT1 mRNA increased in macrophages compared to monocytes of the mouse liver in response to a high fat, high cholesterol diet [18,19]. Some studies have been showed free fatty acids can regulate ACAT1 gene expression [20,21]. This experiment attempt defined the role of fatty acids on the PPARα and ACAT1 gene expression.

Effects of fatty acids on the macrophages and foam cells is vary complicate. Some studies have shown that n-3 and CLA fatty acids are potent anti-atherogenic nutrients but others have been showed the pro-atherogenic potential of CLA [22]. Thus, it is necessary to elucidate the molecular basis of the controversial anti-atherogenic potential of CLA. Therefore study evaluates the role of PPARα, ACAT1 and fatty Acids on the lipid metabolism in foam cells.

## Materials and methods

### Materials

THP-1 cell lined were obtained from the Iranian branch of Institute Pasture, Phosphate buffered saline (PBS) tablets were obtained from Takara, Japan. Cell culture media, Glutamine, penicillin and streptomycin media supplement, Serum Free Medium (SFM) and fetal bovine serum (FBS) were obtained from Invitrogen Corporation, Paisley, UK. phorbol 12-myristate13-acetate (PMA), dimethyl sulfoxide (DMSO), Wy 14643, Sandoz 58-035, linolenic acid, ecosapantaenoic acid and conjugated linoleic acid were obtained from Sigma-Aldrich, USA. Reagents and kits for RNA extraction and reverse transcription were obtained from Qiagen, USA. SYBER Green PCR Master Mix Reagent and RT^2 ^qPCR Primeres (ACAT1, PPARα, β-Actin) purchased from (SuperArray Bioscience Corpotation, USA). All other chemicals were obtained from Sigma Aldrich, USA.

### Cell culture

Human monocytic THP-1 cells were grown in RPMI 1640 medium supplemented with 10% heat inactivated fetal bovine serum (FBS), streptomycin, amphotricin B, sodium pyruvate, 2 mM L-glutamine, 50 μM 2-mercaptoethanol in a humid atmosphere containing 5% CO_2 _at 37°C. For experiments cells were cultured a 1 × 10^6 ^density into 25 cm^2 ^flasks serum free media(SFM) supplemented with 0.25% FBS free albumin.

### Isolation and acetylating of LDL

Isolation of LDL (1.035 g/mL<d< 1.065 g/mL) was achieved after a single ultracentrifugation 40,000 g for 10 h at 4°C in a swinging bucket rotor[23]. Isolated was acetylated by saturated sodium acetate and acetate anhydride was acetylated[24]. Acetylation of LDL confirmed by acetate cellulose electrophoresis (data not shown).

### Cell differentiation and fatty acid treatment

For differentiation THP-1 to macrophage, THP-1 cells were washed with serum free RPMI 1640 and resuspended in SFM in the presence of 100 ng/ml phorbol 12-myristate 13-acetate (PMA) for 72 h [25].

All fatty acids and synthetic ligands dissolved in DMSO(the medium final concentration was ≤ 0.1%). Cells pre-treated with fatty acids and chemical ligands before cholesterol loading for 24 h and then to induction foam cell transformation, cells were incubated with 50 μg/ml Ac-LDL in SFM medium for 48 h[26]. The concentration of fatty acid and chemical ligands in this experiment was: 100 μM LA, EPA, CLA, 50 μM Wy14643, 5 μM sandoz 58-035.

### RNA extraction and Real time quantitative RT-PCR analysis

Expression levels of RNA transcripts were quantitated by real-time PCR. Total RNA was extracted from THP-1 cells using the RNA easy kit (Qiagen, USA). The concentration of RNA was determined by absorbance at 260 nm in relation to absorbance at 280 nm. RNA was stored at -70°C until real-time polymerase chain reaction (PCR) was performed. 1 μg of RNA for real time PCR, reversed transcribed by quantiTect Reverse Transcription Kit (Qiagen, USA) and according to the manufacturer's protocol.

The Real Time PCR was performed by the SYBER Green PCR Master Mix Reagent. Direct detection of polymerase chain reaction (PCR) product was monitored by measuring the increase in fluorescence caused by the binding of SYBER Green to double-stranded DNA. The cycling parameters was 95°C for 10 min, 40 cycles at 95°C for 15 s; 60°C for 60 s. The housekeeping β-Actin transcript was used to normalized for the amount and quality of the RNAs.

### Toxicology assay

**Figure 1:**
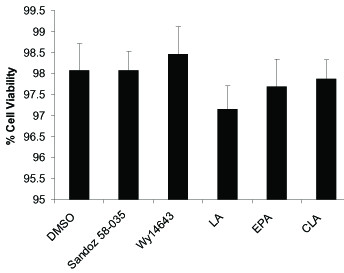
**Cell viability in the presence of fatty acids and synthetic ligands**. Cell viability was determined by the XTT assay and expressed as percentage of absorbance. Data represent mean +SD of at least three experiments run in triplicates. Data showed the fatty acid and other treatments had no significant effect (*P *= 0.118) on cellular viability.

The viability of cells that treatment with 100 μM fatty acid, 50 μM Wy 14643 and 5 μM sandoz 58-035 was quantified spectrophotometrically by the 2, 3-bis (2-methoxy-4-Nitro-5-sulfophenyl)-S-|(phenylamino)carbonyl|-2-tetrazolium hydroxide (XXT) assay (Sigma-Aldrich) as described by Scudiere et al [27]. THP-1 macrophages were cultured with or without the fatty acids and pharmacological ligands for 36 h in 96-well microplate. After an appropriate incubation (37°C, 4 h) with XTT the plates were mixed and absorbance at 570 nm was measured. Results were expressed as percentage of cell viability with respect to control negative absorbance (100% cell viability). XTT assay showed LA, CLA, EPA and synthetic ligands have no effect on the cell viability in THP-1 in comparison with control group (Figure 1).

### Cellular cholesterol measurement

Differentiated THP-1 macrophages cultured in SFM and pre-treated with fatty acids and synthetic ligands for 24 h. Then cells treated with 50 μg/ml Ac-LDL and incubated in the presence of fatty acids or others ligands for 48 h. After this incubation period, cells were washed twice with Phosphate Buffer Saline (PBS) and homogenized in 200 μl hexane-isopropanol (3:2) [28]. The organic phase used for TC and EC measurement by enzymatic assays (Calbiochem, USA). FC was measured as the difference between TC and EC. Cell were lysed with RIPA buffer (10 mM sodium phosphate, 150 mM NaCl, 0.5% sodium deoxycholate, 0.1% SDS, 100 mg/ml PMSF, 30 ml/ml aprotinin and 1 mM sodium orthovanadate, 50 mg/ml Dithiotrithol pH 7.4) [29] and cellular proteins measured by Bradford assay[30]. Results were presented as μg lipids/mg cellular proteins.

### Statistical analysis

All results are presented as means ± S.D. Differences between the groups were determined by one-way ANOVA, with Post-Hoc comparison by Tukeymultiple comparison test. The level of significance for all statistical analyses was set at *P *< 0.05. Analysis was performed using SPSS for Windows software.

## Results

### Cellular EC, FC in Cholesterol-Loaded Macrophages

**Figure 2:**
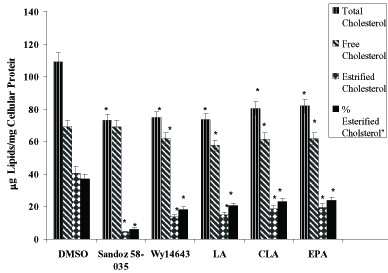
**Fatty acids decreases Intracellular total cholesterol (TC), free cholesterol (FC) and esterified cholesterol (EC) levels in THP-1 derived foam cells**. THP-1 derived macrophages were cholesterol-loaded with Ac-LDL (50 μg/mL) for 48 hours. Wy14643 (50 μmol/L) and Sandoz58-035 (5 μM) was added 24 hours before cholesterol-loading and thereafter every 24 hours. TC and FC were enzymatically determined and CE were calculated as the difference between TC and FC. Results are the mean ± SD of triplicate determinations, representative of 3 independent experiments. Statistically significant differences between treatments are indicated by one-way ANOVA followed by Tukey multicomparison test. Compared with controls all treatments were significant (p ≤ 0.05).

To determine the effect of fatty acids and synthetic ligands(Wy14643, Sandoz 50-035) on the cholesterol distribution between FC and EC, THP-1-derived differentiated macrophages loaded with Ac-LDL (50 μg/mL) for 48 hours. Fatty acids and specific synthetic PPARα ligand (Wy14643) and ACAT1 ligand (Sandoz 50-035) added 24 hours before cholesterol loading. As presented in figure 2 incubation with fatty acids caused a significant decrease in intracellular total cholesterol(TC), free cholesterol(FC), percentage of cellular EC and estrified cholesterol (EC)concentrations (P = 0.05). All of the fatty acids, PPARα activation by Wy14643 decreased cellular TC in foam cells. However inhibition of cholesterol estification by Sandoz 58-035 had no significant effect on the cellular FC (Figure 2).

### Molecular markers of macrophage cholesterol metabolism

**Figure 3:**
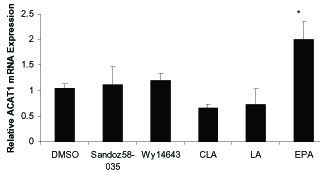
**The effects of fatty acids (100 μM), Wy14643 (50 μmol/L) and Sandoz58-035 (5 μM) on mRNA levels of ACAT1 in THP-1 macrophages derived foam cells**. Cells were treated for 48 h with fatty acids and pharmacological compounds. mRNA levels analyzed by cyber green procedures. The values normalized to β-actin. All results represent means ± SD from triplicate determinations, representative of 3 independent experiments compared with control. Significant differences between treatments are indicated by one-way ANOVA followed by Tukey multicomparison test. *P ≤ 0.05.

**Figure 4:**
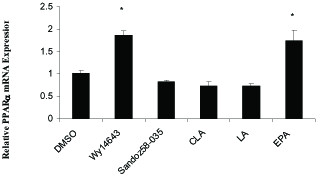
**The effects of fatty acids (100 μM) and pharmacological PPARα and ACAT1 ligands on mRNA levels of PPARα in THP-1 macrophages derived foam cells**. Cells were treated for 48 h with fatty acids and pharmacological compounds. mRNA levels analyzed by cyber green procedures. The values normalized to β-actin. All results represent means ± SD from triplicate determinations, representative of 3 independent experiments compared with control. Significant differences between treatments are indicated by one-way ANOVA followed by Tukey multicomparison test. *P ≤ 0.05.

Figure 3 and figure 4 show the effect of LA, CLA and EPA on PPARα and ACAT1 mRNA expression, in comparison with the PPARα agonist WY-14643 and ACAT1 inhibitor Sandoz58-035.

ACAT1 mRNA expression was significantly increased by 100 μM EPA (P = 0.003) but c9, t11-CLA, LA and Sandoz 58-035 had no significant effect on the ACAT1 mRNA expression compared to foam cells treated with DMSO (control). Treatment with Wy14643 and EPA increased PPARα mRNA levels (P = 0.05, *P *= 0.05 respectively), although CLA and LA had no significant effect on PPARα mRNA expression (figure 4).

## Discussion

Foam cell macrophages are involved in all stages of atherosclerosis, also human THP-1 monocyte-macrophages previously established as a valuable model for studying the lipid metabolism in human macrophages and have been used to study the effects of natural and pharmacological ligands involved in lipid metabolism of macrophage.

Increased uptake of modified LDL, via the scavenger receptors caused the accumulation of unesterified, or free, cholesterol (FC) and esterified cholesterol (EC) by such foam cell macrophages [31].

Pervious studies have shown that n-3 and CLA fatty acids can inhibit the progression and promoting process of atherosclerosis and so are potent anti-atherogenic nutrients in vivo. In contrast Mondy, et al showed the pro-atherogenic potential of CLA in C57BL/6 mice [22]. Thus, it is necessary to elucidate the molecular basis of the controversial anti-atherogenic potential of CLA. Therefore the present study focused on the effects of the individual n-3 fatty acids and CLA on molecular markers of cholesterol homeostasis in THP-1 derived macrophages as the most important component of the process of atherosclerosis.

This study investigated the effects of CLA, LA and EPA fatty acids on the intracellular lipid distribution and ACAT1 and PPARα gene expression in THP-1 derived foam cells. Incubation of THP-1 cells with the different fatty acids and pharmacological showed that intracellular TC, EC, FC and percentage of EC reduced. However Sandoz 58-035 hadn't significant effect on the intracellular FC. Theses data confirm the result obtained by Weldon et al [25] and Chinetti et al [26]. These effects explained by enhanced foam cell cholesterol efflux by increasing the availability of FC for efflux through the ABCA1 and inhibition ACAT1 pathways. Because these fatty acids can effect LXR and SREBP pathways, further works are required to determine the roles of them in lipid metabolism of foam cells.

One of the most important changes in foam cell formation and so initiation and progress of atherosclerosis is accumulation of lipid droplet in macrophages. Many genes and factors are involved id lipid accumulation in macrophage, PPARα and ACAT1 have key role in this phenomenon. One important group of ligands of these factors are fatty acids [25,32]. Many studies showed that differentiation of THP-1 cells into macrophages produced a 2-4-fold increase in ACAT1 expression [32], but some studies reported no increased in ACAT1 mRNA levels in THP-1 cells [33] and others showed that fatty acid derivatives reduced levels of ACAT1 expression [23].

Seo et al and others researchers suggested despite of in fact FFAs can alter ACAT1 mRNA levels in a cell-specific manner; these fatty acids may affect ACAT1 mRNA stability that leads to various levels of ACAT1 mRNA expression. Furthermore, differential ACAT1 regulation by different FFAs could modify storage and distribution of cholesterol in cell [20]. In current study we found that incubation THP-1 derived foam cells with EPA can increase expression of ACAT1 but CLA and LA didn't significant effect.

Another important gene in lipid metabolism in foam cells is PPARα. PPARα is an important lipid-activated transcription factor that has many roles in regulation genes involved in lipid and glucose metabolism and inflammation processes. PPARα is expressed in many types of human cells, such as macrophages as well as in atherosclerotic plaques macrophages. In macrophages, PPARα regulates the balance between free cholesterol and cholesteryl esters in THP-1 derived foam cells [25].

Activation of PPARα by natural or synthetic ligands can induce expression of enzymes such as CPT-1 (carnitine palmitoyltransferase 1), a key enzyme in mitochondrial fatty acid catabolism, and so reduced availability of fatty acids as substrate for ACAT1[26] which can lead to reduction of intracellular TC and EC[26,34]. But it is likely that the stimulation of cholesterol efflux to the plasma membrane by other factors such as genes regulated by LXR results in a reduced intracellular cholesterol as substrate for ACAT1, thus leading to a reduction in the intracellular TC and EC content[35].

Our study showed Wy14643 and EPA increased the levels of PPARα mRNA but CLA and LA didn't effect on the PPARα gene expression. Many experiments showed dietary fatty acids did not induce significant changes PPARα gene expression in THP-1 derived macrophages and foam cells and other tissue [25,36-38]. But some studies found that high polyunsaturated fatty acid diet can increased mRNA levels of PPARα [39].

In current study all fatty acids compare with macrophages increased levels of PPARα mRNA (data didn't present) but compare with foam cells only EPA increased its levels (figure 5), however LA and CLA didn't significant effect. This can showed lipid metabolism in foam cells is different from macrophages and interaction and integration between ligands and others factors in two type's cells are not similar.

## Conclusion

In conclusion it seems that different fatty acids are ligands that have variable affinity for transcription factors and others factors. These ligands have independent effect on the lipid metabolism in foam cells. In the other side coordination between genes involved in foam cell lipid metabolism is very complicated and this is can explain the different results obtained by various researchers.

## Competing interests

The authors declare that they have no competing interests.

## Authors' contributions

ESEand MHcarried out the molecular genetic studies, participated in the sequence alignment and drafted the manuscript. MP carried out the immunoassays. MS participated in the sequence alignment. JZand MM participated in the design of the study and performed the statistical analysis. MDconceived of the study, and participated in its design and coordination. All authors read and approved the final manuscript
