# Modulation Peroxisome Proliferators Activated Receptor alpha (PPAR α) and Acyl Coenzyme A: Cholesterol Acyltransferase1 (ACAT1) Gene expression by Fatty Acids in Foam cell

**DOI:** 10.1186/1476-511X-8-47

**Published:** 2009-10-26

**Authors:** Javad Zavvar Reza, Mahmoud Doosti, Masoud Salehipour, Malehieh Packnejad, Majed Mojarrad, Mansour Heidari

**Affiliations:** 1 Department of Medical Biochemistry, Faculty of medicine, Tehran University of Medical Scenses, Tehran, Iran; 2 Department of Medical Biochemistry, Faculty of medicine, Shahid Sadoughi University of Medical Sciences, Yazd, Iran; 3 Advanced Technologies for novel therapeutics, Newark, USA

## Correction

Following publication of this work [1] Dr Emamian has requested to be be removed as an author as although she saw the first draft she did not see the second and did not approve the work for publication. She also specifically requested not to be included as an author. An apology is extended to Dr Emamian. The Authors Contributions section has been modified as below.

## Authors' contributions

MH carried out the molecular genetic studies, participated in the sequence alignment and drafted the manuscript. MP carried out the immunoassays. MS participated in the sequence alignment. JZ and MM participated in the design of the study and performed the statistical analysis. MD conceived of the study, and participated in its design and coordination.
